# A gene signature predicting prognosis of patients with lower-grade gliomas receiving temozolomide therapy

**DOI:** 10.1007/s12672-023-00818-9

**Published:** 2023-11-13

**Authors:** Yanzhi Wan, Guangqi Li, Junyue Deng, Hong Zhu, Xuelei Ma

**Affiliations:** 1 Department of Biotherapy, Cancer Center and State Key Laboratory of Biotherapy, West China Hospital, Sichuan University, Chengdu, Sichuan China; 2 Department of Medical Oncology, West China Hospital, Sichuan University, Chengdu, 610041 Sichuan China; 3 West China School of Medicine, West China Hospital, Sichuan University, Chengdu, 610041 China

**Keywords:** Lower-grade gliomas (LGGs), Temozolomide (TMZ) therapy, Genes, Risk scores, Prognosis

## Abstract

Temozolomide (TMZ) has been used as a first-line therapy against lower-grade gliomas (LGGs) combined with other chemotherapy drugs. However, there has been no reliable index predicting TMZ response of patients with LGGs. In this study, we aim to investigate the relationship between gene expressions and the prognosis of TMZ therapy in LGGs. We integrated transcriptome and clinical data of 171 LGGs from the Chinese Glioma Genome Atlas (CGGA). Consensus LASSO Cox regression was used to identify 14 key genes related to different clinical outcomes under TMZ chemotherapy. We constructed and evaluated a risk score based on the 14 genes. Patients with LGGs of lower risk scores (low-risk group) generally had better survival than those LGGs of higher risk scores (high-risk group), which is independent of clinicopathological factors. High-risk patients showed activation of innate and humoral-type immunity. The prognostic contribution of the risk score was validated in an independent validation cohort of 65 patients. Besides, combined with three independent predictors (grade, IDH1 mutation status, and chr1p19q co-deletion status), we further developed a nomogram to predict the benefit of TMZ treatment in LGGs. Our results indicate that a transcriptome-based index can optimize the treatment strategy for patients with LGGs under TMZ therapy.

**Supplementary Information:**

The online version contains supplementary material available at 10.1007/s12672-023-00818-9.

## Introduction

Gliomas, the most common primary tumors of the central nervous system, are grouped by the World Health Organization (WHO) with histopathology from grade I (lowest grade) to grade IV (highest grade) [1, 2]. The higher grade indicates a higher malignancy. Lower-grade gliomas (LGGs), a diverse group of grade II and III gliomas, generally have higher treatment effectiveness and a better prognosis in comparison with glioblastoma (grade IV). However, LGGs, incurable diseases, have a high risk of recurrence because of the characteristics of diffuse growth and the potential for progression [4, 5]. The median overall survival for LGG patients ranges from 2 to 15 years based on many factors, such as tumor histopathological, molecular phenotype, growth rate, and therapeutic strategy [6].

Nowadays, Temozolomide (TMZ) is used as the first-line therapy against LGGs combined with other chemotherapy drugs. Because of its lipophilicity, Temozolomide (TMZ) can easily penetrate the blood–brain barrier [7]. It shows good tolerability with limited side effects, thus becoming an effective drug for the treatment of glioma [8]. However, the therapeutic efficacy of TMZ remains very limited due to its frequent resistance in glioma. Besides, treatment effectiveness differs among patients receiving the same therapeutic strategy. Some data already suggested a close connection between gene mutations, malignant transformation, and TMZ response [9, 10]. Some previous studies managed to portray the clinical behavior of LGGs related to genomic mutation. Other studies constructed a prognostic model using biological features (DNA repair ability as well as cell cycle processes) that are crucial to tumor recurrence [7, 11, 12]. Yet the relationship between genes and the prognosis of patients receiving Temozolomide (TMZ) adjuvant chemotherapy has been left undiscovered. It remains a challenge to effectively assess the prognosis of lower-grade gliomas.

The Chinese Glioma Genome Atlas (CGGA) project includes the long-term follow-up data of LGG patients with TMZ treatment, providing the possibility for the establishment of treatment prognosis scores. In this study, we gathered a large number of samples to identify the connection between genes and the prognosis of patients receiving TMZ, therefore confirming that prognosis scores, consisting of some key genes, are indeed related to the prognosis of TMZ therapy. Our results provide further evidence of assessing the Prognosis of TMZ treatment through key genes for the better benefit of patients.

## Methods

### Data sources

We curated the clinical and gene expression data of LGG patients from the Chinese Glioma Genome Atlas. The first batch of data from the CGGA database was used as a training set to develop TMZ treatment prognosis scores, and the second batch of data from the CGGA database was used to evaluate the prognostic performance of risk scores. Only patients with complete overall survival time and transcriptomics data were retained. The gene expression data were log2- transformed for subsequent analysis.

### Prognostic gene analysis

The Cox regression was used to screen genes associated with the prognosis of TMZ treatment after correcting significant clinical variables. Genes with p values less than 0.01 were considered to be potentially associated with treatment prognosis. Then these genes were then classified as protective or risky according to whether HR was less than 1. Gene set enrichment pathway analysis was conducted based on Gene Ontology (GO), Kyoto Encyclopedia of Genes and Genomes (KEGG), and Reactome database by using the online tool Metascape. The protein–protein interaction network analysis was carried out by the STRING database, and only the high-confidence edges were retained. The Molecular Complex Detection (MCODE) algorithm has been applied to identify densely connected network components. Pathway enrichment analysis was also applied to each MCODE component independently, and the three best-scoring terms by p-value have been retained as the functional description of the corresponding components.

### Establishment of treatment prognosis scores

These genes which were potentially associated with treatment outcomes were subjected to penalized multivariate Cox proportional hazards survival modeling using an algorithm for variable selection based on L1-penalized (Lasso) estimation. A penalty parameter, λ1, reflecting the predictive potential and calculated by cross-validation, was inflicted upon the gene expression signals during survival modeling. Using this model, some key genes were selected via tenfold cross-validation in the training sets. Due to the randomness of cross-validation, the selected key genes were variable, so we used consensus Lasso regression to identify key genes. That is, genes retained at high frequency after multiple run Lasso regression can be considered to be the most influential for treatment prognosis. Using the regression modeling strategies (RMS) packages to draw nomograms and decision curves. Decision curve analysis (DCA) was performed utilizing the DCA package.

The order of frequency represented the degree of influence of these genes, and then these genes were incorporated into the Cox model in turn. We then used the area below the time-dependent ROC to evaluate the prognostic efficacy of the model. When the ROC reaches a certain point, the model is optimal and contains the least genes, which will be identified as the key genes for prognosis. lasso is highly dependent on seed when it is allowed. The order of frequency represents the degree of influence of these features, and then these features are incorporated into the COX model in turn, and the inclusion stops when AUROC reaches its peak, at which time the model is optimal and contains the least features. Finally, we used tenfold cross-validation in the training set to determine the weight coefficients of these genes, and the cumulative sum of the product of gene weight and corresponding expression was used as the TMZ prognosis score. And the clinical variables were pooled and a visualized nomogram was drawn using R package rms.

### Evaluation of treatment prognosis scores

We systematically assessed the prognostic efficacy of risk scores in training sets and independent validation sets. Patients were firstly divided into high- and low-risk groups using the median risk score as the cutoff. Then the Kaplan–Meier method was used to draw the survival curves of the two groups, and the log-rank test was used to judge the difference. The prediction accuracy was evaluated by time-dependent AUC obtained by R package timeROC. We used calibration curves to assess the agreement between nomogram-predicted risks and observed risks and performed decision curve analyses to measure the net benefit of identifying truly high-risk patients who should intervene because of their use of nomogram as a screening tool.

### Statistical analysis

R (V 4.1.3) software was used for the above statistics and analysis. The images were stitched together by Adobe Illustrator software. Wilcoxon rank-sum test was used for the comparison of box plots. The Spearman coefficient was used for correlation analysis. The Chi-square test (Fisher's exact test if necessary) was used to compare the clinical features of the two groups. Multivariate logistic regression analysis was used to evaluate the clinical features that affected clustering. Kaplan Meier method was used to plot the survival curve. Cox analysis was used to assess characteristics associated with survival. All hypothesis tests were two-sided tests. P-values of multiple tests were corrected by the FDR method.

## Results

### Analysis of genes related to the prognosis of LGG patients after TMZ treatment

**Fig. 1:**
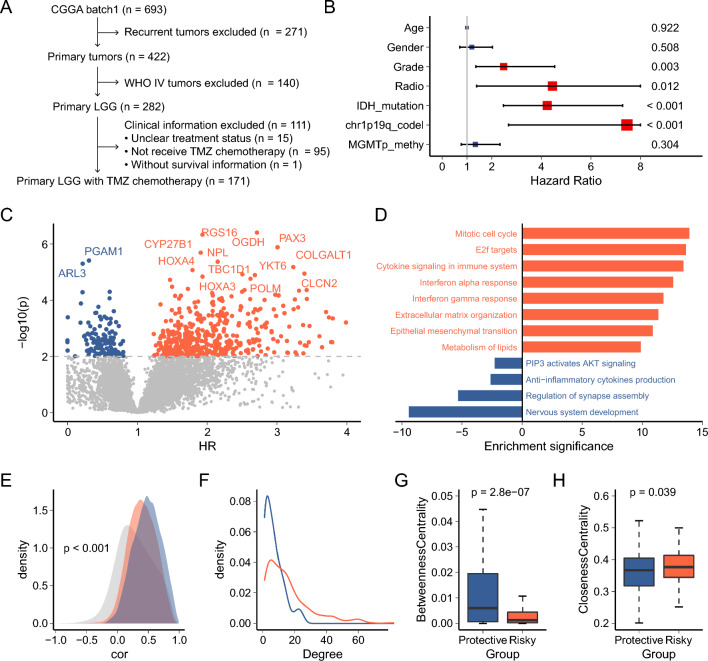
Analysis of genes related to the prognosis of LGG patients after TMZ treatment. **A** A total of 171 patients were used to analyze factors associated with TMZ prognosis in lower-grade gliomas. **B** Association of patient clinical information with TMZ outcome. Univariate Cox regression is used for analysis and significant associations are represented in red. **C** Genes associated with TMZ prognosis after correction for clinically relevant variables. Analyses were performed using multivariate COX regression, and significant protective and risk factors are shown in blue and orange, respectively. **D** Functions associated with protective and risk genes, and blue and orange represent protective and risky functions, respectively. **E** Distributions of associations between protective, risk, and other genes. Blue, orange, and gray represent protective, risk, and other genes, respectively. **F** Degree distribution in PPI networks formed by protective and risk genes. The blue and orange lines represent protective and risky PPI networks, respectively. **G** Differences in betweenness centrality of nodes between PPI networks formed by protective and risk genes. **H** Differences in closeness centrality of nodes between PPI networks formed by protective and risk genes

We obtained 171 lower-grade gliomas (LGG) patients receiving temozolomide (TMZ) chemotherapy from the CGGA database as a training set to screen for genes associated with TMZ treatment prognosis (Fig. 1A). Meanwhile, we obtained 65 lower-grade gliomas (LGG) patients receiving temozolomide (TMZ) chemotherapy from the CGGA database as an independent validation set (Supplementary Fig. 1). There is some relevant basic clinical information such as histology, grade, gender, age, overall survival, IDH_mutation_status, 1p19q_codeletion_status, and MGMTp_methylation_status of patients in the training set (Supplementary Table 1) and validation set (Supplementary Table 2).

Univariate Cox regression showed that tumor stage, radiation therapy status, *IDH1* mutation status, and chr1p19q co-deletion status had an impact on overall survival (Fig. 1B, Supplementary Fig. 2). Therefore, we used Cox regression to correct these clinical variables to obtain genes associated with the prognosis of TMZ treatment. A total of 488 risk genes and 128 protective genes passed the screening threshold (p < 0.01; Fig. 1C, Supplementary Table 3). Functional enrichment of these genes revealed that protective genes were associated with nervous system development, while risk genes were associated with multiple pathways associated with tumor progression including cell cycle, immune response, EMT, and extracellular matrix organization (Fig. 1D, Supplementary Table 4).

We further analyzed the internal correlations of risk genes, protective genes, and non-significant genes, and found that the overall correlations of protective genes and risk genes were higher than those of non-significant genes, suggesting that these genes formed specific functional modules (Fig. 1E). At the same time, the overall correlation of protective genes was significantly higher than that of risk genes, suggesting that risk genes were involved in multiple functions. In addition, we analyzed the protein network formed by risk and protective genes respectively. The degree distribution of the two networks showed that the network of risk genes reflected multi-modularity (Fig. 1F). Moreover, the centrality measures of the two networks are different in distribution. The mean betweenness centrality of the network of risk genes was low (Fig. 1G), and the mean closeness centrality was high (Fig. 1H). Network module analysis revealed that risk genes formed multiple functional modules, including lipid metabolism, cell cycle, interferon response, and Wnt signaling pathway (Supplementary Fig. 3, Supplementary Table 5). These results suggest that TMZ prognostic risk genes are involved in various carcinogenic processes.

### Development of risk score associated with the benefit of TMZ treatment in LGG

**Fig. 2:**
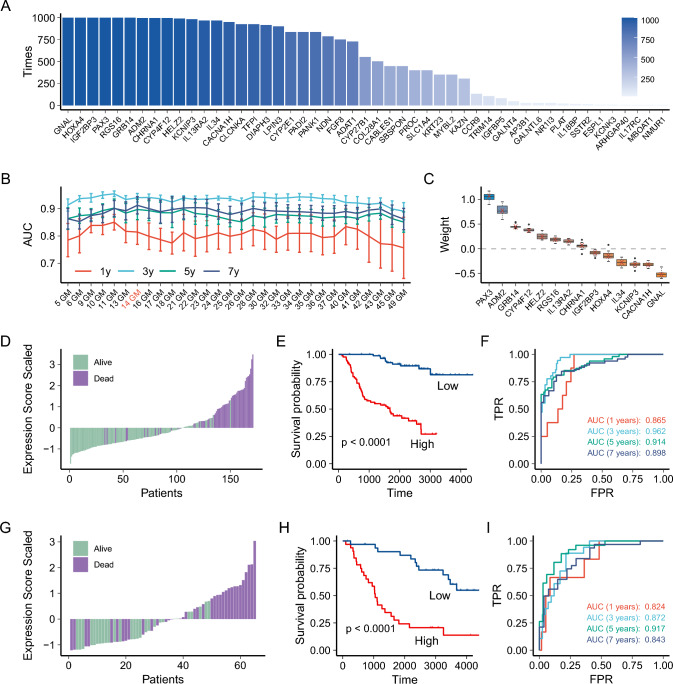
Development of risk score associated with the benefit of TMZ treatment in LGG. **A** The occurrence frequency of each gene in consensus LASSO Cox regression. **B** The area under time-dependent ROC for predicting survival of different gene models (GM) in the training set. **C** The weight of each gene across the tenfold cross-validation. Boxes show median, first, and third quartile, with whiskers extending at most 1.5 interquartile range, and the red Cross indicates the represent the estimated weights for each gene. **D** Association of risk scores with patient survival status in the training set. **E** Differences in survival time between groups with high and low-risk scores in the training set. **F** AUC values for risk scores predicting survival in the training set. **G** Association of risk scores with patient survival status in the validation set. **H** Differences in survival time between groups with high and low-risk scores in the validation set. **I** AUC values for risk scores predicting survival in the validation set

We used consensus LASSO Cox regression to screen key genes for prognostic genes and repeated 1000 iterations to obtain the occurrence frequency of each gene (Fig. 2A), of which 14 genes appeared more than 950 times. We then included high-frequency genes in the model in turn and used a tenfold cross-validation method to evaluate the prognostic model performance by the area under time-dependent ROC at varying follow-up times. The results showed that the prognostic model had the highest predictive power when predicting 3 year survival, while its predictive power was relatively poor for 1 year survival (Fig. 2B). Based on the overall prediction of different survival conditions, we selected a model consisting of 14 genes. For these 14 genes, we used Cox regression to determine the contribution of these genes based on a tenfold cross-validation method. These features, ranked by median weight, included *PAX3* (1.06), *ADM2* (0.76), *GRB14* (0.44), *CYP4F12* (0.37), *HELZ2* (0.24), *RGS16* (0.19), *IL13RA2* (0.15), *CHRNA1* (0.06), *IGF2BP3* (−0.08), *HOXA4* (−0.15), *IL34* (−0.28), *KCNIP3* (−0.32), *CACNA1H* (−0.33), and *GNAL* (−0.52) (Fig. 2C). Finally, the cumulative sum of the product of gene weight and corresponding expression was used as the TMZ prognosis score.

**Fig. 3:**
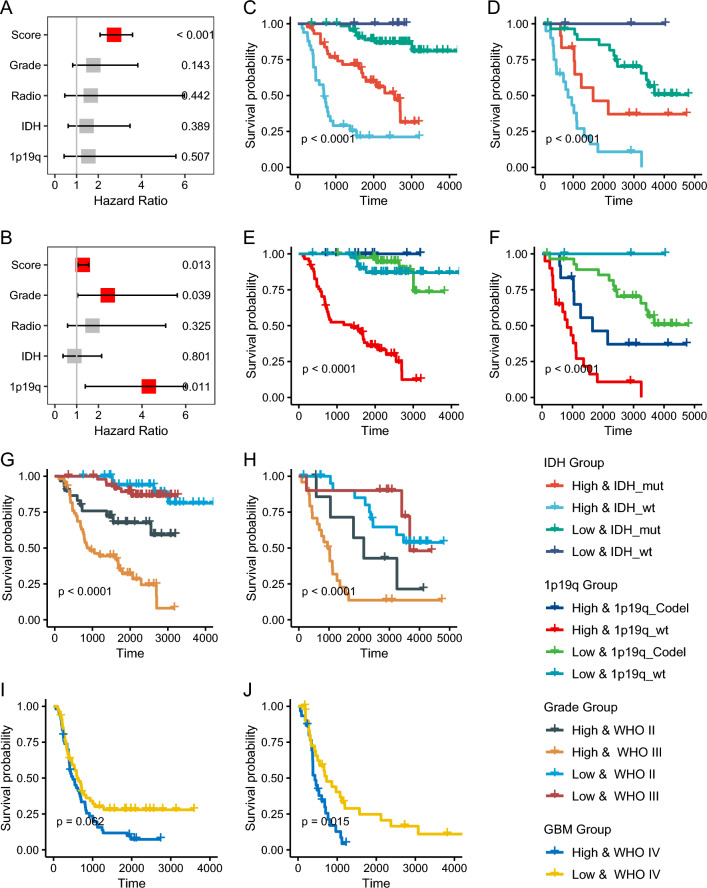
The prognostic power of risk scores was independent of clinicopathological factors. **A** Multivariate Cox analysis of risk scores with multiple clinical factors in the training set. **B** Multivariate Cox analysis of risk scores with multiple clinical factors in the validation set. **C** Effect of IDH status combined with risk score on survival in the training set. **D** Effect of IDH status combined with risk score on survival in the validation set. **E** Effect of chr1p19q co-deletion status combined with risk score on survival in the training set. **F** Effect of chr1p19q co-deletion status combined with risk score on survival in the validation set. **G** Effect of WHO grade combined with risk score on survival in the training set. **H** Effect of WHO grade combined with risk score on survival in the validation set. **I** Differences in survival curves between high and low-risk scores in GBM in CGGA1 datasets. **J** Differences in survival curves between high and low-risk scores in GBM in CGGA2 datasets

After multivariable adjustment by clinicopathological variables including grade, radio status, IDH mutation status, and chr1p19q co-deletion status, the risk score remained a powerful and independent factor in the training cohort (HR 2.73, 95% CI 2.09–3.58, p < 0.001; Fig. 3A). We also noted similar results in the independent validation cohort (HR 1.28, 1.05–1.55, p = 0.013; Fig. 3B). The *IDH1* mutation status and chr1p19q co-deletion status are key genomic markers related to the prognosis of glioma treatment. When stratified by *IDH1* mutation status, the risk score was still a clinically and statistically significant prognostic model in the train and independent validation cohort (Fig. 3CD). We got a similar conclusion, in which patients with chr1p19q wild-type and the high-risk score had the worst survival when patients were stratified according to the co-deletion status of chr1p19q (Fig. 3EF). We also focused on the prognostic effect of tumor stage on risk score, and the risk score can distinguish patients with different stages from two groups with significant differences in survival (Fig. 3GH). Further, we explored the prognostic performance of risk scores in glioblastoma (GBM). Although there are certain differences between LGG and GBM in the genome and other aspects, we found that the risk score still showed certain prognostic ability, and patients with lower risk scores tended to have better survival benefits (p = 0.062 in train data sets and p = 0.015 in independent validation sets; Fig. 3IJ).

### The risk score was related to the immune cell infiltration of LGG

**Fig. 4:**
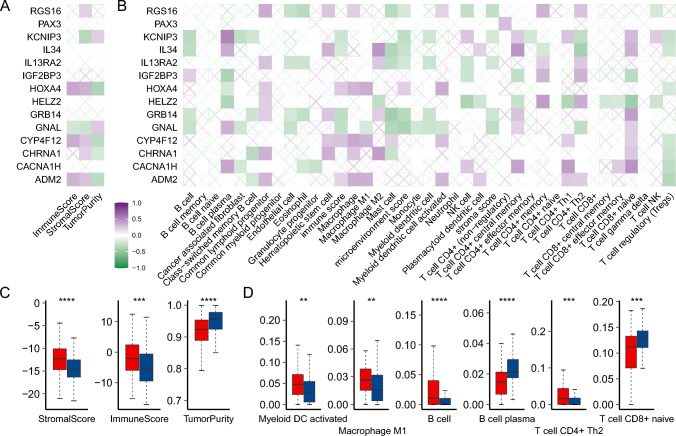
The risk score was related to the immune cell infiltration of LGG. **A** The relation between genes in the model and immune score from the ESTIMATE method. **B** The relation between genes in the model and immune score and percentage of immune cell infiltration from the Xcell method. **C** Differences in tumor purity, immune score, and stromal score between high and low-risk groups. **D** Differences in immune cell infiltration between high and low-risk groups. Red represents the high-risk score group and blue represents the low-risk score group

We then analyzed the relationship between risk score and the immune microenvironment. We use a variety of algorithms including ESTIMATE, XCELL, and CIBERSORT to measure the composition of the tumor's immune microenvironment. We first analyzed the association of key genes in the model with tumor purity, immune score, stromal score, and different immune cell infiltration proportion. The *HOXA4*, *CYP4F12*, and *ADM2* were positively correlated with the immune and stromal score but negatively correlated with tumor purity (Fig. 4A). The immune cells that are positively associated with these genes include macrophages, naïve CD8 + T cells, and activated dendritic cells (Supplementary Fig. 4, Fig. 4B).

The *GNAL*, on the other hand, was negatively correlated with the immune score, stromal score, and some myeloid lymphocyte infiltration (Fig. 4AB). We then assessed differences in immune microenvironments between high and low-risk groups. Patients with high-risk scores had higher immune and stromal scores and lower tumor purity (Fig. 4C), indicating that unfavorable prognosis in the high-risk group may be associated with the variation in the tumor immune microenvironment. Further immune cell infiltration analysis showed that the activated dendritic cells, M1 type macrophages, B cells, and Th2 subset of CD4 + T cells were higher in patients with high-risk scores, while the B cell plasma and naïve CD8 + T cells were lower in patients with high-risk score (Supplementary Fig. 5, Fig. 4D). These results indicated that high-risk patients showed activation of innate and humoral-type immunity.

### Development of a nomogram for predicting the benefit of TMZ treatment in LGG

**Fig. 5:**
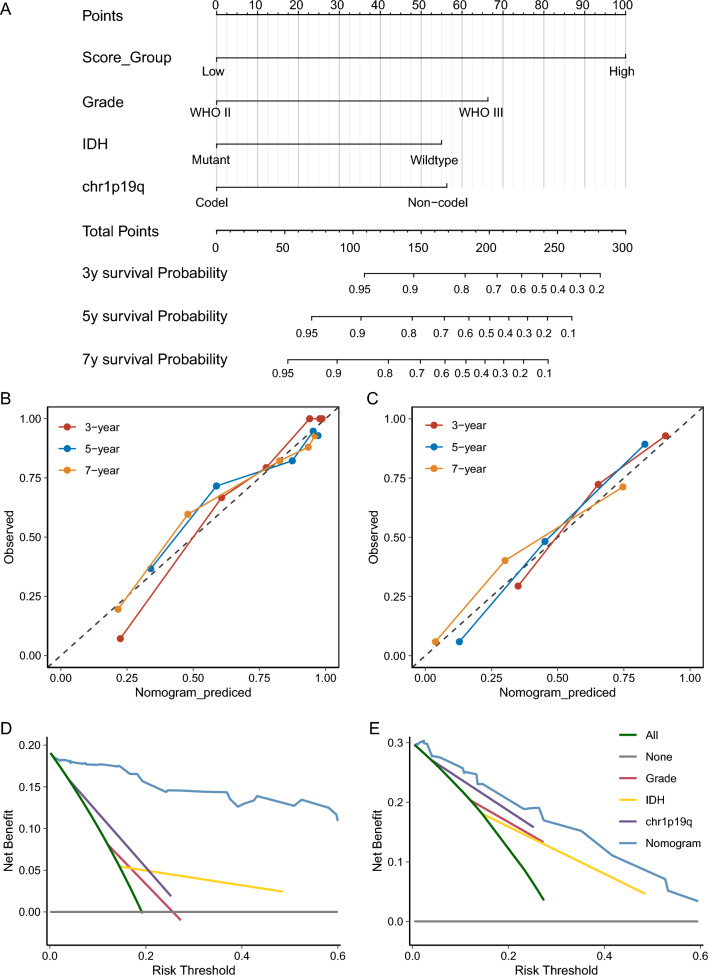
Nomogram of risk score. **A** A nomogram based on risk score, grade, IDH status, and chr1p19q co-deletion status. **B** Calibration curves of nomogram in the training set. **C** Calibration curve analysis of nomogram in the training set. **D** Decision curves of nomogram in the validation set. **E** Decision curve analysis of nomogram in the validation set

Med well in predicting patient survival according to an ideal model in training and independent validation sets (Fig. 5BC). To evaluate the clinical benefit and better predict the prognosis of LGG patients after receiving TMZ therapy in the clinic, a prognostic nomogram was developed by integrating risk score and three independent predictors of mortality including grade, *IDH1* mutation status, and chr1p19q co-deletion status from the above analyses into a multivariate Cox regression model (Fig. 5A). The calibration plot showed that the nomogram perforators of the nomogram model, we performed decision curve analysis in both the training and independent validation sets. With 3 year survival as the endpoint, the curve showed that the nomogram presented more clinical net benefits than several competing intervention strategies, namely, intervention for all, intervention for none, and intervention based on different clinical indicators (Fig. 5DE).

## Discussion

Temozolomide chemotherapy is the main treatment for LGGs. However, most patients are prone to developing drug resistance during the treatment process, and its therapeutic effect varies greatly among different patients. Biological heterogeneity among patients with LGGs contributes to dramatically different treatment outcomes. Hence, we systematically assess the influence of transcriptomes on the prognosis after Temozolomide chemotherapy, attempting to find the relationship between some key genes and prognosis.

Tumor stage, radiation therapy status, IDH1 mutation status, and chr1p19q co-deletion status, these elements all have been identified as important factors related to patients’ overall survival. Therefore, we managed to find other associated factors that are independent of clinicopathological features. Genes associated with TMZ treatment prognosis are emerging into our sights. In our study, we classified the genes into risk genes, protective genes, and non-significant genes. The protective genes were associated with nervous system development, while risk genes were associated with tumor progression into higher grades. Finally, we identified 14 key genes from 488 risk genes and established the TMZ-prognosis score, which was the cumulative sum of the product of gene weight and corresponding expression.

We identified a risk score that was associated with the prognosis of Temozolomide therapy in LGGs. The numerical of risk scores were related to the prognosis of patients–patients with lower risk scores generally had better survival and significant differences were shown in survival time between the low and high gropes. Two large randomized EORTC studies already attempted to predict the prognosis of LGGs through risk scores, which include 5 factors. Patients with a low-risk prognostic score (< two factors) generally had longer survival than those with a high-risk score(3–5 factors) [13].

Relevant research has explored the predictive genes related to the Temozolomide sensitivity of glioblastomas [14]. However, no research has figured out the relationship between key genes and TMZ-related prognosis in lower-grade gliomas. Our result fills the gap. We found that 14 keys genes, including PAX3 (1.06), ADM2 (0.76), GRB14 (0.44), CYP4F12 (0.37), HELZ2 (0.24), RGS16 (0.19), IL13RA2 (0.15), CHRNA1 (0.06), IGF2BP3 (− 0.08), HOXA4 (− 0.15), IL34 (-0.28), KCNIP3 (− 0.32), CACNA1H (− 0.33), could form a TMZ prognosis score. Which could help us to better predict the treatment effect and outcome of the patients. A higher risk score in LGG patients often has a worse prognosis. These key genes play crucial roles in LGG development. PAX3 is overexpressed in glioblastomas [15] and it contributes to features of glioma stem cells, which has been identified as a diagnostic/prognostic marker and a therapeutic target [16]. GRB14 plays glioma-promoting roles through PDGFRα, which promotes glioma progression and treatment resistance [17]. RGS16 has been related with CD8 + T cell exhaustion (PMID: 35622904). Higher expression of RGS16 is significantly correlated with poor prognosis of patients with gliomas [18]. HELZ2, ADM2 are also related with prognosis of various types of cancer [19–24]. Some key genes may affect patients’ prognosis by modulating TMZ drug sensitivity. For example, CYP4F12 encodes enzymes of the cytochrome P450 superfamily, which are related with TMZ metabolism [25]. We found that for the high-risk group, median survival was 1680 days; while not reach for the low-risk group. What’s more, we suggested that this module had better performance in 3- and 5 year survival predictions than 1- or 7 year survival predictions, although the AUCs of survival years at different times are all above 0.8. Besides, these key genes may not only predict the prognosis of TMZ in treating glioma but also help us identify potential molecular markers for TMZ sensitivity/resistance even chemotherapy targets [14, 26]. Some regulatory elements could even modulate glioma cell sensitivity to temozolomide through long-range regulation of multiple target genes.

However, for some LGG patients with low-risk scores, we were unable to accurately evaluate their survival years. Whether can we incorporate more genes to solve this problem, is a challenge need to face. As we know, an earlier clinical stage means a better prognosis. Data suggests that LGG patients without 1p and 19q co-deletion means better responses to chemotherapy and longer overall survival [27]. Radiation therapy status is also an important factor, which has a great influence on overall and progression-free survival times [28]. Although it has no predictive value for chemotherapy response, IDH1 mutation is an independent prognostic factor in LGGs [29, 30]. The prognostic power of risk scores was independent of the above-related clinicopathological factors. However, data from other messages suggests that besides those elements, prognosis also depends on other elements like age at diagnosis, Karnofsky performance status (KPS), first presenting symptom, extent of resection, and certain molecular markers [31]. There is one limitation of this study that should be noted here. In our results, because of limited data from the CGGA database, we only make more influential factors as independent factors, while some little influential factors are not taken into account. Our risk scores may perform better in mostly LGGs patients, but they may have accurate predictions for some patients with early age or basic diseases.

Interestingly, we found an association between risk score and immune microenvironment, suggesting the indirect effect of the immune microenvironment on prognosis. High-risk patients are more likely to show activation of innate and humoral-type immunity, which may cause a worse prognosis. Besides, to better predict the prognosis of LGG patients after receiving TMZ therapy in the clinic, a prognostic nomogram was developed, which integrates risk score and three independent predictors of mortality including grade, IDH1 mutation status, and chr1p19q co-deletion status into a multivariate Cox regression model. It provides comprehensive and individualized analysis of different patients to assess the prognosis. We would like to provide free online software for patients and healthcare providers.

In summary, we not only found an association between key genes and prognosis but also established a valuable risk scores tool for LGGs after receiving Temozolomide chemotherapy. This tool provides an individualized estimate of survival based on the specific genes of different patients. By predicting the prognosis of patients, clinicians can better design follow-up modes and optimize treatment to improve survival outcomes. To facilitate the clinical use of this nomogram, free online software for its implementation will be provided.

### Supplementary Information

Additional file 1 (XLSX 54 KB)

Additional file 2 (XLSX 30 KB)

Additional file 3 (XLSX 62 KB)

Additional file 4 (XLSX 204 KB)

Additional file 5 (XLSX 10 KB)

Additional file 6 (DOCX 965 KB)

## Data Availability

The datasets presented in this study can be found in online repositories. The names of the repository/repositories and accession number(s) can be found in the article/Supplementary Material.
