# Supplementary material for: A gene signature predicting prognosis of patients with lower-grade gliomas receiving temozolomide therapy
Source: Discov Oncol. 2023 Nov 13;14:202. doi: 10.1007/s12672-023-00818-9 (PMC10643648; doi:10.1007/s12672-023-00818-9)
Supplement: Supplementary file 6 — Additional file 6 (DOCX 965 KB) [file 12672_2023_818_MOESM6_ESM.docx]

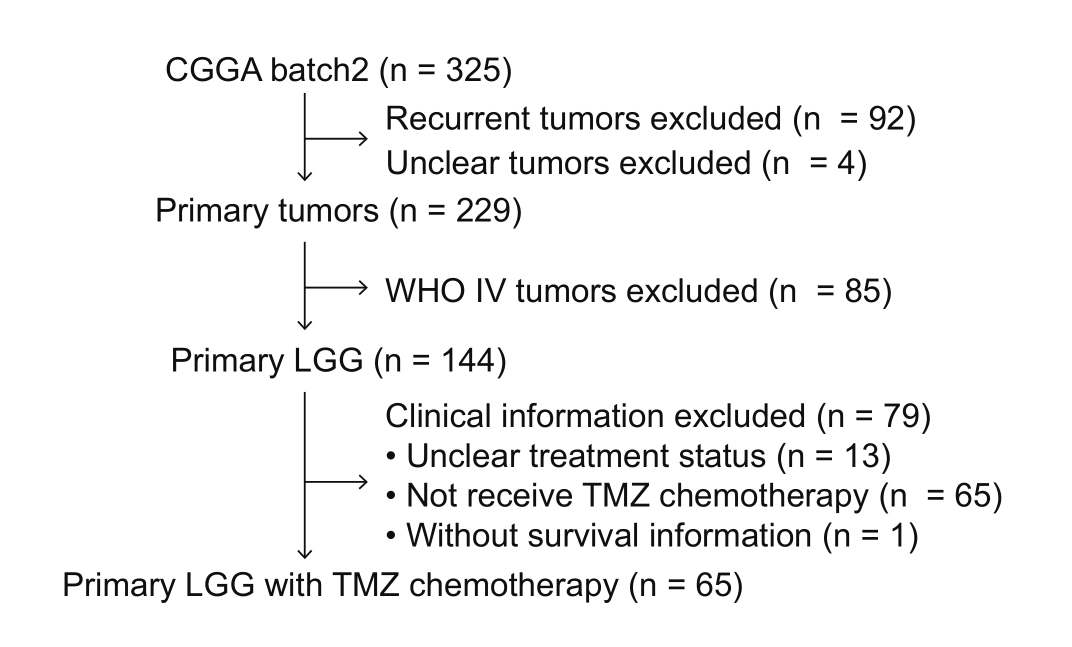


**Supplementary Figure 1.** A validation set to screen for genes associated with TMZ treatment prognosis.


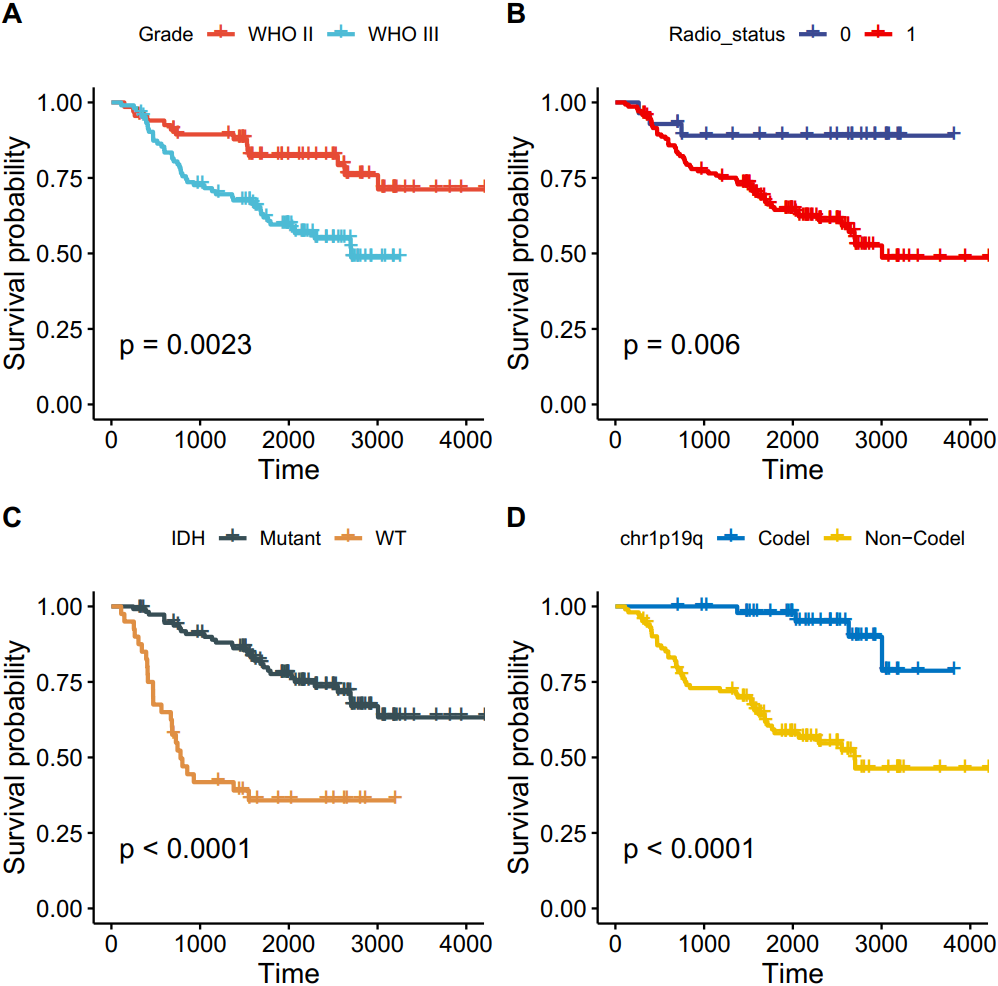


**Supplementary Figure 2.** Survival analysis of clinical factors associated with prognosis of TMZ treatment.


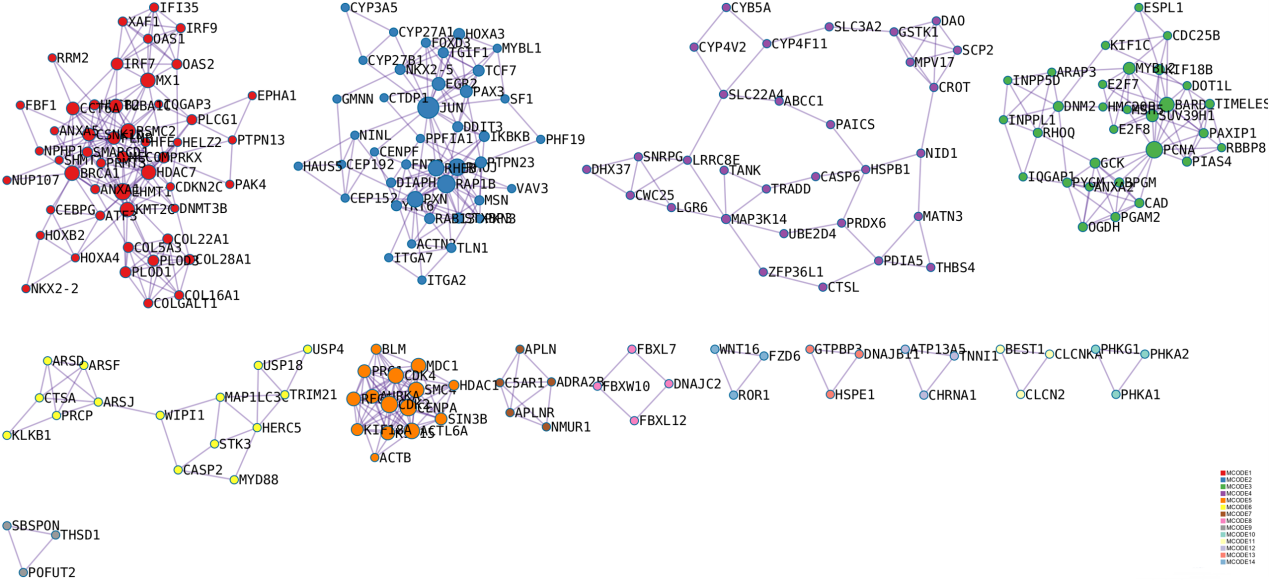


**Supplementary Figure 3.** Network module analysis for risk genes. Different colors represent different modules.


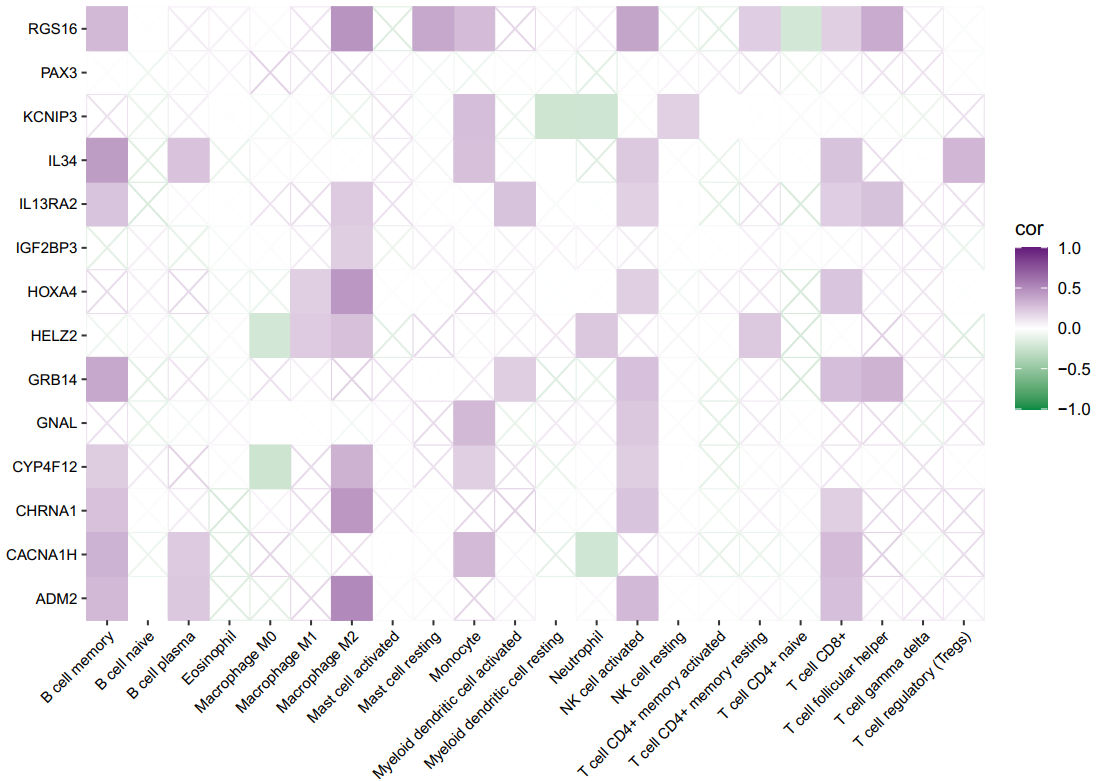


**Supplementary Figure 4.** The relation between genes in the model and immune score and percentage of immune cell infiltration from the CIBERSORT method.


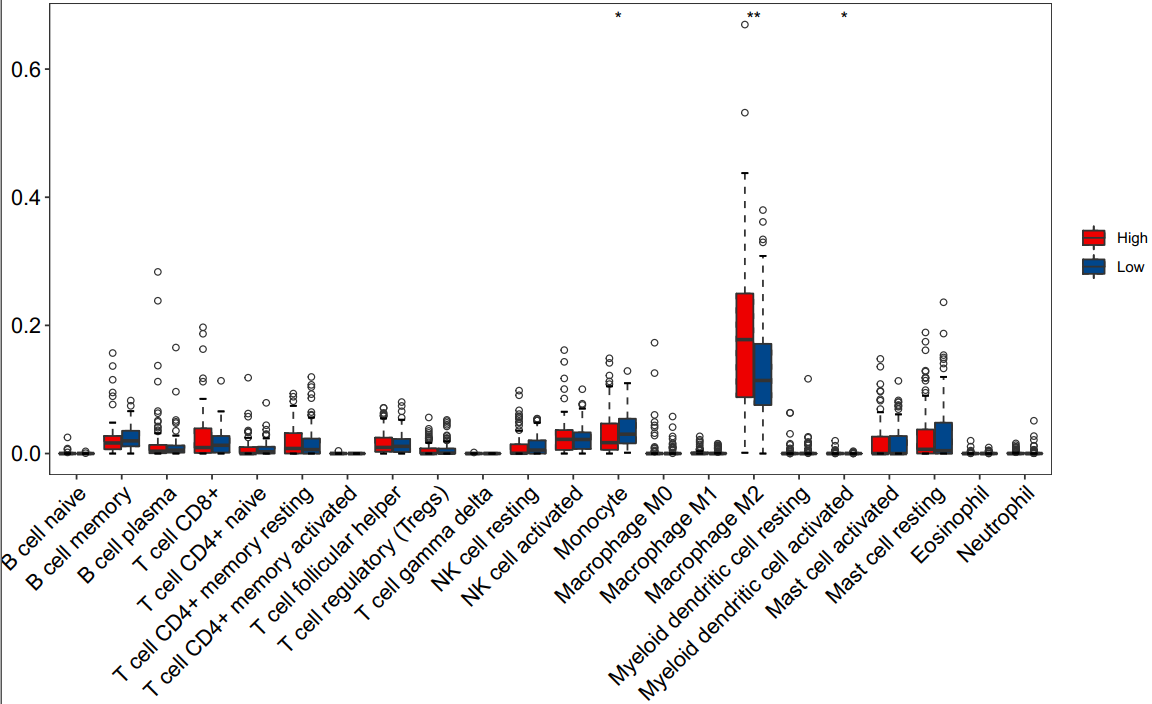


**Supplementary Figure 5.** Differences in immune cell infiltration from the CIBERSORT method between high and low-risk groups. Red represents the high-risk score group and blue represents the low-risk score group.
